# Applications of CRISPR/Cas9 in the Synthesis of Secondary Metabolites in Filamentous Fungi

**DOI:** 10.3389/fmicb.2021.638096

**Published:** 2021-02-11

**Authors:** Chunmiao Jiang, Gongbo Lv, Yayi Tu, Xiaojie Cheng, Yitian Duan, Bin Zeng, Bin He

**Affiliations:** ^1^Jiangxi Key Laboratory of Bioprocess Engineering and Co-Innovation Center for In-Vitro Diagnostic Reagents and Devices of Jiangxi Province, College of Life Sciences, Jiangxi Science and Technology Normal University, Nanchang, China; ^2^College of Life Sciences, Sichuan Normal University, Chengdu, China; ^3^School of Information, Renmin University of China, Beijing, China; ^4^College of Pharmacy, Shenzhen Technology University, Shenzhen, China

**Keywords:** CRISPR-Cas9, genome editing, secondary metabolites, filamentous fungi, CRISPRa

## Abstract

Filamentous fungi possess the capacity to produce a wide array of secondary metabolites with diverse biological activities and structures, such as lovastatin and swainsonine. With the advent of the post-genomic era, increasing amounts of cryptic or uncharacterized secondary metabolite biosynthetic gene clusters are continually being discovered. However, owing to the longstanding lack of versatile, comparatively simple, and highly efficient genetic manipulation techniques, the broader exploration of industrially important secondary metabolites has been hampered thus far. With the emergence of CRISPR/Cas9-based genome editing technology, this dilemma may be alleviated, as this advanced technique has revolutionized genetic research and enabled the exploitation and discovery of new bioactive compounds from filamentous fungi. In this review, we introduce the CRISPR/Cas9 system in detail and summarize the latest applications of CRISPR/Cas9-mediated genome editing in filamentous fungi. We also briefly introduce the specific applications of the CRISPR/Cas9 system and CRISPRa in the improvement of secondary metabolite contents and discovery of novel biologically active compounds in filamentous fungi, with specific examples noted. Additionally, we highlight and discuss some of the challenges and deficiencies of using the CRISPR/Cas9-based genome editing technology in research on the biosynthesis of secondary metabolites as well as future application of CRISPR/Cas9 strategy in filamentous fungi are highlighted and discussed.

## Introduction

Filamentous fungi have a major impact on many aspects of human diets and health more broadly. Their benefits are derived from various mechanisms underlying the production of enzymes, organic acids, and flavors, as well as, more importantly, antibiotic compounds and therapeutic molecules (Satish et al., 2020). Moreover, they are well known as appealing microbial cell factories that possess the industrial capability to secrete a large repertoire of different bioactive secondary metabolites, such as paclitaxel and swainsonine, which have become important clinical therapeutics (Hautbergue et al., 2018; Keller, 2019; Mosunova et al., 2020). There has been a focus on research into secondary metabolites because some secondary metabolites can be used as anti-cancer drugs and anti-bacterial compounds (Lobanovska and Pilla, 2017). A majority of these secondary metabolites can be classified into three chemical categories: polyketides derived from acyl-CoAs, terpenes produced from acyl-CoAs, and small peptides derived from amino acids (Keller, 2019; Mosunova et al., 2020). Most of the genes involved in the biosynthesis of secondary metabolites are frequently clustered together on chromosomes in biosynthetic gene clusters (SM-BGCs), and some are not expressed under standard laboratory culture conditions (Lin et al., 2013; Jiang et al., 2015; Nah et al., 2017; Keller, 2019; Kjaerbolling et al., 2019). Another characteristic of genes in such clusters is that they are not constitutively expressed, and formerly actively expressed genes can become transcriptionally quiescent upon repeated culturing (Kale et al., 1994; Hoffmeister and Keller, 2007). Although secondary metabolite biosynthetic genes in filamentous fungi are generally found in clusters that provide a convenient genetic locus for manipulation (Hoffmeister and Keller, 2007), the exploitation of new bioactive compounds is hindered by intrinsic difficulties involving complex genetic backgrounds and poor efficiency of gene targeting. With increases in genomic insight and gene mining from high-throughput sequencing data and the advancement of genomics and transcriptomics, there has been an acceleration in the identification and utilization of SM-BGCs. Thus, elucidating the genetic basis and the biosynthetic pathways of secondary metabolites has become comparatively easier.

Filamentous fungi, such as *Trichoderma reesei*, *Aspergillus niger*, *Aspergillus oryzae*, and *Aspergillus nidulans*, are universally used as model eukaryotic microorganisms to produce industrial secondary metabolites. For example, researchers have utilized rice blast fungus *Pyricularia oryzae* to yield tenuazonic acid, *Aspergillus fumigatus* to secrete the natural product trypacidin, *Fusarium fujikuroi* to produce gibberellic acid, *Fusarium heterosporum* to synthesize the polyketide equisetin, *A. nidulans* to secrete microperfuranone, and *Penicillium chrysogenum* to biosynthesize sorbicillin (Gauthier et al., 2012; Kakule et al., 2015; Yun et al., 2017; Shi et al., 2019). However, while the genetic manipulation of secondary metabolites in filamentous fungi is being explored, there are several factors that limit genetic research on secondary metabolite biosynthetic pathways of filamentous fungi. First, filamentous fungi, like the model organism yeast, have complex genetic backgrounds when compared to prokaryotes (Song et al., 2019). Second, it is difficult to apply genetic manipulation and molecular biology tools in filamentous fungi. Furthermore, low homologous recombination efficiency (generally less than 5%) and a lack of suitable selection markers, and plasmids also hamper the exploitation of novel secondary metabolites from filamentous fungi (Mei et al., 2019).

More recently, a variety of genetic engineering methods, such as RNA interference, heterologous expression, gene-targeting strategies, and zinc finger nuclease (ZFN) and transcription-activator-like effector nuclease (TALEN) – based genome editing have been developed to explore and demonstrate the biosynthetic and regulatory mechanisms in filamentous fungi (Boettcher and McManus, 2015; Wang et al., 2017; Dasgupta et al., 2020). Although the existing approaches can be utilized to edit target genes at the genomic level, these do not meet the needs of industrial secondary metabolite production in filamentous fungi owing their low editing efficiency and cumbersome manipulation (Shi et al., 2017). Thus, the exploitation of new secondary metabolites with potential pharmaceutical applications in filamentous fungi is extremely challenging. Fortunately, the emergence of the clustered regularly interspersed short palindromic repeats (CRISPR)/associated protein (Cas) system in recent years has raised hopes of solving the problem presented by the largely inefficient gene editing tools available for use in filamentous fungi. The current CRISPR/Cas systems were discovered in archaea and bacteria and can be classified into three group based on the different Cas effectors (Cas9, Cas13, and Cas12), which can then be further divided into six types and more than 20 subtypes (Makarova et al., 2018; Li et al., 2019). The type-II CRISPR/Cas system from *Streptococcus pyogenes* has been widely applied across species, including filamentous fungi, as it is much simpler than other CRISPR systems and has proven to be particularly powerful for use in precise DNA modification (Cong et al., 2013; Deng et al., 2017b). Using specific codon optimization and *in vitro* RNA transcription, Liu et al. (2015) first adopted the CRISPR/Cas9 system in the filamentous fungus *T. reesei* and achieved relatively high homologous recombination efficiencies (>93%) when the lengths of the homology arms were 200 bp. In the same year, Nodvig et al. (2015) successfully targeted the *yA* gene by applying this system in the model fungus *A. nidulans* and obtained a genome-edited phenotype. Additionally, Fuller et al. (2015) demonstrated that the CRISPR/Cas9 system can be applied to high-efficiency gene disruption in *A. fumigatus*. These instances illustrate that this powerful system has been widely and effectively applied to industrial filamentous fungi. More details about the CRISPR/Cas9 system and its specific application in the biosynthesis of secondary metabolites by filamentous fungi are reviewed in the following sections.

This review introduces and summarizes the current knowledge and applications of the CRISPR/Cas9 system in filamentous fungi. By detailing several examples, we introduce the specific application of the CRISPR/Cas9 system and CRISPRa for precise gene editing and gene cluster activation, respectively. Additionally, we discuss and summarize the challenges and limitations as well as further prospects of this technology in the production of secondary metabolites by filamentous fungi. Our review lays a solid foundation for the exploration of secondary metabolites in filamentous fungi and will be beneficial to future research on activating silent gene clusters involved in secondary metabolites produced by filamentous fungi.

## Principle and Advantages of the CRISPR/CAS9 System

**FIGURE 1:**
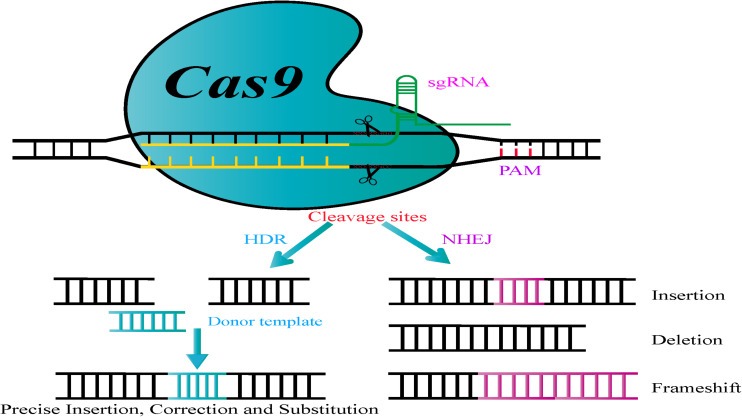
Principle of the CRISPR/Cas9 genome engineering tool. Nuclease-induced double-stranded breaks (DSBs) can be repaired by non-homologous end joining (NHEJ) or homology-directed repair (HDR) pathways. Imprecise NHEJ-mediated repair can produce insertion/deletion/frameshift mutations of variable lengths at DSB sites. Homologous recombination (HR)-mediated repair can introduce precise point mutations, by substitutions, insertions, or deletions, depending on the donor DNA template.

The CRISPR/Cas9 system that has emerged as an advanced technology for genome engineering originated from adaptive immune systems in bacteria as a special defense mechanism against invading viruses and plasmids (Barrangou and Marraffini, 2014; Wang and Coleman, 2019). This genome editing system is composed of two components, a Cas9 nuclease and a guide RNA molecule (gRNA) that targets the nuclease to a specific genomic target site in the genome (Deng et al., 2017b). The single chimeric guide RNA (sgRNA) consisting of a fusion of a CRISPR RNA (crRNA) and a fixed *trans-*activating crRNA (tracrRNA) are processed by the endogenous bacterial machinery to generate the mature gRNA (Deltcheva et al., 2011). The Cas9 endonuclease is guided to a specific locus by a gRNA, which then forms Watson-Crick base pairs with the target DNA sequence, thereby permitting Cas9 to break the double-stranded DNA at specific sites (Figure 1; Doudna and Charpentier, 2014; Mei et al., 2019). Importantly, to achieve the complementary target-DNA binding and cleavage, Cas9 requires the presence of its major specificity determinant, a well-defined short protospacer adjacent motif (PAM) that is located immediately adjacent to the non-target DNA strand (Mojica et al., 2009; Sternberg et al., 2014). Subsequently, two unique repair mechanisms (Figure 1), non-homologous end-joining (NHEJ) or homology-directed repair (HDR), mend DNA double-strand breaks (DSBs). NHEJ is an error-prone and dominant RNA repair pathway for DSBs via direct ligation of the break ends without using a homologous template. Thus, it can sometimes cause targeted mutations, such as random deletions, insertions, replacement of bases, targeted chromosomal rearrangements, or frameshift mutations at DNA breakage points leading to premature stop codons within the open reading frame (ORF) of the targeted gene (Kim and Kim, 2014; Boettcher and McManus, 2015; Mei et al., 2019). Compared to NHEJ, which is the most common DSB repair mechanism in microorganisms, HDR is a less efficient but high-fidelity pathway. HDR precisely repairs DSBs with the help of a homologous DNA template or exogenous donor fragment, thus having the potential to generate gene modifications by introducing desired nucleotide substitutions or gene insertions (Bortesi and Fischer, 2015; El-Sayed et al., 2017). With the advancement of research and accumulating knowledge on endogenous DNA repair mechanisms, a series of technological tools have been explored to precisely induce DSBs in target genes by using exogenous nucleases. Prior to CRISPR, genome engineering strategies utilizing ZFN or TALEN-based genome editing required the design, generation, and validation of an appropriate protein for a specific DNA locus of interest, thus limiting their widespread application (Doudna and Charpentier, 2014; Boettcher and McManus, 2015; El-Sayed et al., 2017; Leitão et al., 2017). Owing to its high efficiency and the possibility of multi-gene editing, the CRISPR/Cas9 system has rapidly emerged as an extraordinary genome engineering approach has outstripped the performance of earlier technologies (Doudna and Charpentier, 2014; Wang et al., 2017).

## Applications of CRISPR/CAS9 in Filamentous Fungi

The versatility and programmability of Cas9 has made the CRISPR/Cas9 genome editing strategy a revolutionary approach in biological research, and it has been considered useful for creating gene deletions, substitutions, and insertions in filamentous fungi. Here, we gather considerable information regarding the application of the CRISPR/Cas9 system in filamentous fungi over recent years (Table 1). Apart from genetic modification, Cas9 can also modulate transcription without editing the genomic sequence by fusing the enzymatically inactive version of Cas9 (dCas9) with transcriptional repression and activation domains (Gilbert et al., 2013, 2014; Chavez et al., 2015). The former approach, fusion of dCas9 with repression domains (e.g., KRAB/Kox1), has been used to negatively regulate the transcription of specific genomic loci (Gilbert et al., 2013; Leitão et al., 2017). This strategy, commonly termed CRISPR inhibition (CRISPRi), decreases the transcription of target DNA loci mainly by blocking transcriptional elongation, impeding transcription factor binding, or interfering with RNA polymerase transcription initiation (Bikard et al., 2013; Larson et al., 2013; Doudna and Charpentier, 2014; Piatek et al., 2015). Several reports have revealed that this approach can be successfully applied to simultaneously repress the transcription of multiple target genes and that it is reversible (Bikard et al., 2013; Gilbert et al., 2013; Zhao et al., 2014). CRISPR activation (CRISPRa), can be achieved through direct fusing dCas9 with activation domains, such as VP64/p65/HSF1 (Bikard et al., 2013; Gilbert et al., 2014; Konermann et al., 2015; Jia et al., 2018; Strezoska et al., 2020). For example, Cheng et al. (2013) achieved robust endogenous gene activation utilizing VP160 transcriptional activation domain in human and mouse cells. With the intention of enhancing transcriptional activation, Chavez et al. (2015) introduced a unique activation strategy that required the fusion of dCas9 to three activation domains, VP64-p65-Rta (VPR) and thus proved its utility in activating endogenous coding and non-coding genes. CRISPRa also has potential in exploring gene expression in filamentous fungi, particularly, for genes that are closely related to secondary metabolite biosynthesis (Wang and Coleman, 2019). For example, Roux et al. (2020) constructed the first CRISPRa system in *A. nidulans* by using the same method as Chavez et al. (2015) to improve production of the compound microperfuranone and identify a new metabolite of dehydromicroperfuranone. Filamentous fungi have the capacity to produce a diverse spectrum of valuable secondary metabolites, and the genetic potential of secreting important metabolites has not yet been fully utilized for most SM-BGCs. Therefore, there is little doubt that a strategy that combines the CRISPR/Cas9 technology (i.e., CRISPRa) with the activation of expression of secondary metabolite biosynthetic clusters is on the horizon. This would be particularly useful in developing bioactive products or derivatives for biopharmaceuticals.

**TABLE 1:** Applications of CRISPR-Cas9 genome engineering tool in filamentous fungi.

Species	Cas9 expression (selection marker, promoter)	Transformation method	Editing method	Application	Reference
*Phytophthora sojae*	Human-optimized codons Cas9, *G418*, *Ham34*	PMT^1^	NHEJ/HDR	Single-gene disruption	Fang and Tyler, 2016
*Ustilago maydis*	Codon-optimized Cas9, *ip*, *Otef* (modified tef1)*/hsp70*	PMT	NHEJ	Single/double-gene disruption	Schuster et al., 2016
*A. fumigatus*	Human codon-optimized Cas9, *pyr4/hph*, *niiA/gpdA*	PMT	HDR	Single/double-gene disruption	Zhang et al., 2016
*A. oryzae*	Codon-optimized Cas9, *pyrG*, *amyB*	PMT	NHEJ	Single-gene disruption	Katayama et al., 2016
*Penicillium chrysogenum*	Human codon-optimized Cas9, *amdS*, *xlnA*	PMT	NHEJ/HDR	Single-gene disruption	Pohl et al., 2016
*Candida albicans*	Codon-optimized Cas9, *Nat*, *ENO1*	Lithium acetate	NHEJ/HDR	Single-gene disruption	Min et al., 2016
*A. fumigatus*	pFC332, *pyrG*, *TetON*	PMT	NHEJ/HDR	Single-gene disruption	Weber et al., 2017
*A. niger*	pFC332, *pyrG/hph*, *tef1*	PMT	NHEJ/HDR	Single-gene disruption	Kuivanen et al., 2016
*Ustilago maydis*	B913, *ip*, *Otef* (*modified tefl*)/ *hsp70*	PMT	NHEJ	Multiple-gene disruption	Schuster et al., 2018
*A. oryzae*	Codon-optimized Cas9, *niaD/pyrG*	PMT	NHEJ	Double-gene disruption	Nakamura et al., 2017
*Myceliophthora thermophila*	Codon-optimized Cas9, *Bar*, *tef1*	PMT/AMT^2^	NHEJ/HDR	Single/multiple-gene disruption	Liu et al., 2017
*Talaromyces atroroseus*	pFC332, *hph*, *tef1*	PMT	NHEJ	Single-gene disruption	Nielsen et al., 2017
*Aspergillus carbonarius*	pFC332, *hph*, *tef1*	AMT	NHEJ/HDR	Single-gene disruption	Weyda et al., 2017
*A. niger*	pFC332, *hph*, *tef1*	PMT	NHEJ	Single-gene disruption	Kuivanen et al., 2017
*Ganoderma lucidum*	Codon-optimized Cas9, *ura3*, *gpdA*	PMT	NHEJ	Single-gene disruption	Qin et al., 2017
*Beauveria bassiana*	Codon-optimized Cas9, *gfp/ura5/bar*, *gpdA*	PMT	NHEJ/HDR	Single/multiple-gene disruption	Chen et al., 2017
*Alternaria alternata*	pFC332, *pyr4/hph*, *gpdA*	PMT	NHEJ	Single-gene disruption	Wenderoth et al., 2017
*Shiraia bambusicola*	Codon-optimized Cas9, *hph*, *TrpC*	PMT	NHEJ	Single-gene disruption	Deng et al., 2017a
*Shiraia bambusicola*	Codon-optimized Cas9, *hph*, *TrpC*	PMT	NHEJ/HDR	Single-gene disruption	Deng et al., 2017c
*Nodulisporium sp.*	Codon-optimized Cas9, *Bar*, *TrpC*	PMT	NHEJ/HDR	Single-gene disruption	Zheng et al., 2017
*Mucor circinelloides*	SpCas9, *pyr4*	PMT	NHEJ/HDR	Single/double-gene disruption	Nagy et al., 2017
*Leptosphaeria maculans*	Human codon-optimized Cas9, *Ip/G418/hph*, *act1*	AMT	NHEJ	Single-gene disruption	Idnurm et al., 2017
*Sporisorium scitamineum*	*HCas9*, *Nat/Hph*, *gapd*	AMT	HDR	Single-gene disruption	Lu et al., 2017
*A. niger*	pCas9, *hyg/pyrG*	PMT	NHEJ	Single-gene disruption	Sarkari et al., 2017
*A. fumigatus*	*In vitro*-assembled Cas9 RNPs, *hyg*	PMT	HDR	Single-gene disruption	Al Abdallah et al., 2017
*Magnaporthe oryzae*	Codon-optimized Cas9, *hyg*	PMT	NHEJ/HR	Single/double-gene disruption	Foster et al., 2018
*M. thermophila*	Codon-optimized Cas9, *neo*, *tef1*	PMT	NHEJ	Single-gene disruption	Xu et al., 2018
*Rhizopus delemar*	Codon-optimized Cas9, *pyrF*	Electroporation	NHEJ	Single-gene disruption	Bruni et al., 2018
*M. thermophila*	Codon-optimized Spmae, *hyg/bar/neo*, *tef1/gpdA/TrpC*	PMT	HDR	Single/multiple-gene disruption	Gu et al., 2018
*A. niger*	Codon-optimized Cas9, *pyrG/ble*, *tRNA*	PMT	HDR	Single-gene disruption	Song et al., 2018
*Penicillium decumbens*	Cas9 RNPs, *gpdA*, *ergA*	PMT	HDR	Single/multiple-gene disruption	Grijseels et al., 2018
*Fusarium graminearum*	Codon-optimized Cas9, *fludioxonil*, *gpdA*	PMT	NHEJ/HDR	Single-gene disruption	Gardiner and Kazan, 2018
*A. niger*	pFC332, *hph*, *tef1*	PMT	NHEJ	Single-gene disruption	Kuivanen and Richard, 2018
*C. albicans*	SpCas9, *Nat/Phloxine B*	Electroporation	NHEJ	Double-gene disruption	Shapiro et al., 2017
*Aspergilli*	pFC332, *argB/pyrG*, *tef1*	AMT	HDR	Multiple-gene disruption	Nodvig et al., 2018
*Blastomyces dermatitidis*	pFC332, *hph*, *tef1*	AMT	NHEJ	Single/double-gene disruption	Kujoth et al., 2018
*A. niger*	Codon-optimized Cas9, *hph/amdS*, *glaA*	PMT	NHEJ/HDR	Single-gene disruption	Zheng et al., 2019
*C. albicans*	Codon-optimized Cas9, *Nat*, *ENO1*	Electroporation	HDR	Single/double-gene disruption	Vyas et al., 2018
*P. sojae*	Human-optimized codons Cas9, *G418*, *Ham34*	PMT	NHEJ	Double-gene disruption	Miao et al., 2018
*F. oxysporum*	pFC332, *hph*	PMT	NHEJ/HDR	Single-gene disruption	Wang et al., 2018
*Ustilaginoidea virens*	Codon-optimized Cas9, *G418*, *pdc/cbh1*	AMT/PMT	NHEJ	Multiple-gene disruption	Liang et al., 2018
*Cordyceps militaris*	Codon-optimized Cas9, *5-FOA/blpR*, *tef1*	AMT/PMT	NHEJ/HDR	Single-gene disruption	Chen et al., 2018
*Cryptococcus neoformans*	Codon-optimized Cas9, *Ntc*, *tef1*	Electroporation	HDR	Single-gene disruption	Wang, 2018
*Sclerotinia sclerotiorum*	Codon-optimized Cas9, *hph*, *tef1*	PMT	NHEJ/HDR	Single-gene disruption	Li et al., 2018
*Aspergillus fumigatus*	pFC332, *pyrG, tef1*	PMT	NHEJ	Single-gene disruption	Matsuda et al., 2018
*Ustilago trichophora*	Human-optimized codons Cas9, *marker-free*, *U6*	PMT	NHEJ	Single-gene disruption	Huck et al., 2019
*Penicillium subrubescens*	Codon-optimized Cas9, *hph*, *tef1*	PMT	NHEJ	Single-gene disruption	Salazar-Cerezo et al., 2020
*A. niger*	pFC332, *hph*, *tef1*	PMT	HDR	Multiple-gene disruption	van Leeuwe et al., 2019
*M. thermophila*	Codon-optimized Cas12a, *neo/bar*, *U6*	PMT	NHEJ/HDR	Multiple-gene disruption	Liu et al., 2019
*A. niger*	pFC332, *argB/pyrG*, *tef1*	PMT	HDR	Single-gene disruption	Leynaud-Kieffer et al., 2019
*A. oryzae*	Codon-optimized Cas9, *pyrG*, *amyB*	PMT	HDR	Single/double-gene disruption	Katayama et al., 2019
*Alternaria alternata*	pFC332, *hph*, *tef1*	PMT	NHEJ	Single-gene disruption	Igbalajobi et al., 2019
*A. niger*	Codon-optimized Cas9, *pyrG*	PMT	NHEJ/HDR	Single/multiple-gene disruption	Kuivanen et al., 2019
*Mucor circinelloides*	SpCas9, *pyr4*	PMT	HDR	Multiple-gene disruption	Nagy et al., 2019
*Leptosphaeria maculans*	Human codon-optimized Cas9, *hph*, *act1*	AMT	NHEJ	Double-gene disruption	Darma et al., 2019
*F. fujikuroi*	Codon-optimized Cas9, *hph*, *U6*/*5SrRNA*	PMT	HDR	Multiple-gene disruption	Shi et al., 2019
*Ashbya gossypii*	Human-optimized codons Cas9, *G418*, *tef1*	Electroporation	NHEJ	Single-gene disruption	Jimenez et al., 2019
*Duddingtonia flagrans*	pFC332, *hph*, *tef1*	PMT	HDR	Single-gene disruption	Youssar et al., 2019
*A. oryzae*	Aspergillus-optimized codons Cas9, *pyrG*, *U6*	PMT	HDR	Single-gene disruption	Chutrakul et al., 2019
*T. reesei*	Codon-optimized Cas9, *pyr4*	PMT	HDR	Single-gene disruption	Hao and Su, 2019
*M. thermophila*	Codon-optimized Cas9, *bar/neo*, *tef1*	PMT	HDR	Double-gene disruption	Li et al., 2020a
*Glarea lozoyensis*	Codon-optimized Cas9, *neo*, *5S rRNA*	AMT	NHEJ/HDR	Single/multiple-gene disruption	Wei et al., 2020
*Talaromyces pinophilus EMU*	Codon-optimized Cas9, *hph*, *gdpA*	PMT	NHEJ	Single-gene disruption	Manglekar and Geng, 2020
*A. niger*	*pCsR1*, *hph*, *tef1*	PMT	NHEJ/HDR	Single/multiple-gene disruption	Rojas-Sánchez et al., 2020
*M. thermophila*	Codon-optimized Cas9, *neo*, *trpC*	PMT/AMT	HDR	Single/multiple-gene disruption	Li et al., 2020b
*A. niger*	pFC332, *pyrG/amdS*, *PnaII/TPI*	PMT	HDR	Single-gene disruption	Dong et al., 2020
*T. reesei*	Codon-optimized Cas9, *ura5*, *U6*	AMT	NHEJ	Single-gene disruption	Wu et al., 2020
*A. niger*	pCas9, *pyrG/hph*, 5S rRNA	PMT	HDR	Double-gene disruption	Zhang et al., 2020

*^1^AMT, Agrobacterium tumefaciens-mediated transformation; ^2^PMT, Protoplast transformation.*

## Specific Application of CRISPR/CAS9 in Secondary Metabolites Synthesis Pathways

In the past few years, the CRISPR/Cas9 system has been introduced into filamentous fungi to explore the potential of this strategy in modulating production of secondary metabolites. From *A. oryzae* and *T. reesei to A. niger* and *A. nidulans*, CRISPR/Cas9 based systems have become versatile platforms for precise genome editing, and great progress has already been made for production of valuable secondary metabolites. Here, we highlight four examples illustrating the application of CRISPR/Cas9 (gene deletion/substitution/insertion) and CRISPRa (gene cluster activation) systems in filamentous fungi.

### Application of CRISPR/Cas9-Based Genome Editing in the Production of Gibberellic Acid by *Fusarium fujikuroi*

Natural products derived from the secondary metabolism of filamentous fungi have a wide array of applications, especially in the pharmaceutical and agricultural industries. Gibberellic acids (GAs) are a class of natural plant growth hormones that are notably produced in *F. fujikuroi* and are widely applied to regulate the growth of diverse plant species. Among all GAs, the most biologically active ones are GA1, GA3, GA4, and GA7 (Ullah et al., 2014). Of these, the production and application of GA3 has reached a particularly mature stage, while that of GA4 and GA7 have been hindered by the low efficiency of the existing production methods (Joshi et al., 2018; Qian et al., 2018).

**FIGURE 2:**
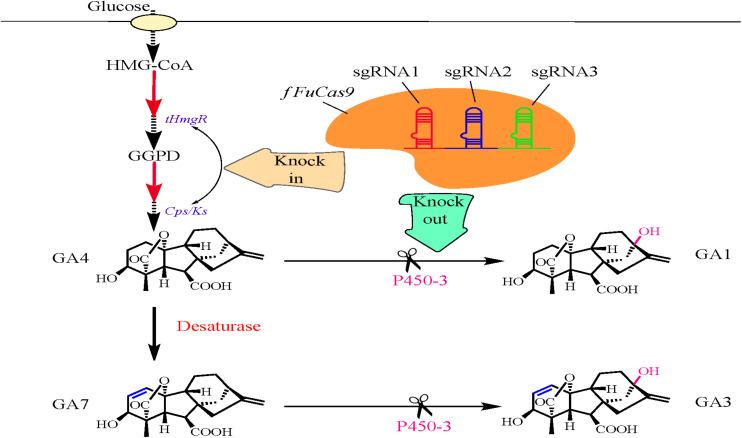
Application of the CRISPR/Cas9-based genome editing tool in *Fusarium fujikuroi* for improving the production of the gibberellic acids GA4 and GA7.

Shi et al. (2019) established an efficient CRISPR/Cas9-based genome editing tool to improve the production of GA4 and GA7 in *F. fujikuroi* (Figure 2). Initially, a *f FuCas9* vector carrying different nuclear localization signals (NLSs) was constructed. The endogenous NLS from histone H2B (HTB_*NLS*_) was selected to individually fuse with the *f FuCas9* protein, owing to its higher editing efficiency and previous success in *Fusarium oxysporum* (Wang et al., 2018). The *f FuCas9* fusion protein vector along with sgRNAs was assembled *in vitro* to establish the CRISPR/Cas9 system (*pUC-f FuCas9-HTB_*NLS*_-hph*). Simultaneously, owing to the need for efficient sgRNA transcription, the endogenous *5S rRNA* (*Ff5SrRNA*) was utilized to express the sgRNA designed for the target gene. Subsequently, the *Ff5SrRNA-P450-3* sgRNA cassette was synthesized with three sgRNAs and ligated into the *EcoRI* site of *pUC-f FuCas9-HTB_*NLS*_-hph*, thus yielding the *pUC-f FuCas9-HTB_*NLS*_-hph-P450-3* vector. This vector was used to disrupt the gene encoding *P450-3*, thus yielding the final disruption mutant Δ*P450-3*. Subsequently, sgRNA and *f FuCas9* vector were introduced into *F. fujikuroi* through the modified protoplast-based polyethylene glycol and Ca^2+^ transformation method (Hwang and Ahn, 2016). Additionally, overexpression of two key genes encoding copalyl diphosphate synthase/kaurene synthase (*Cps/Ks*) and a truncated HMG-CoA reductase (*tHmgR*) were also performed separately and simultaneously to explore the possibility of increasing the amount of GA4/GA7 mixture produced based on previous research (Albermann et al., 2013). To overexpress *Cps/Ks* and *tHmgR* genes, the *f FuCas9* vector and donor vector were introduced by protoplast transformation into the Δ*P450-3* disruption mutant. The resulting transformants were subjected to a second round of hygromycin screening and a series of cultivation cycles and finally inoculated into the fermentation medium. The contents of four GAs in the supernatant fraction of the fermentation medium were determined by high-performance liquid chromatography (HPLC). Compared with the control strain (88.38 mg/L), the accumulation of a GA4/GA7 mixture was evidently improved in the Δ*P450-3* mutant (410.27 mg/L). Thus, the CRISPR/Cas9-based genome editing system proved to be efficient enough to enhance the levels of secondary metabolites in *F. fujikuroi*. Thus, overexpression of both *Cps/Ks* and *tHmgR* has been demonstrated to be an effective mean of increasing the contents of GA4 and GA7 (by 24.23% to 509.68 mg/L and by 70.14% to 698.03 mg/L, respectively), which is clearly higher than in the Δ*P450-3* disruption mutant. Indeed, the combined concentration of GA4 and GA7 reached a higher level (716.37 mg/L) when *Cps/Ks* and *tHmgR* were overexpressed simultaneously in the Δ*P450-3* mutant. Thus, Shi et al. (2019) successfully demonstrated that CRISPR/Cas9-based genome editing strategies and overexpression approaches are suitable for improving the content of GA4/GA7 mixtures. These results will also greatly facilitate further research on production of other metabolites in *F. fujikuroi*.

### Functional Reconstitution of Trypacidin Biosynthesis Gene Cluster in *A. fumigatus* by the CRISPR/Cas9-Based Approach

*Aspergillus fumigatus* is an important human pathogen responsible for various forms of aspergillosis in humans as well as in animals (Paulussen et al., 2017). It also possesses the capacity to secrete a large repertoire of natural products, some of which are involved in pathogenicity (Scharf et al., 2014). Trypacidin, one of the natural antimicrobial antibiotic compounds produced by *A. fumigatus*, is a spore-borne product that has been demonstrated to be a potent toxin to human lung cells (Gauthier et al., 2012). This compound may also be a virulence determinant that plays a role in the phagocytosis of different cells such as murine alveolar macrophages and the amoeba *Dictyostelium discoideum* (Mattern et al., 2015).

**FIGURE 3:**
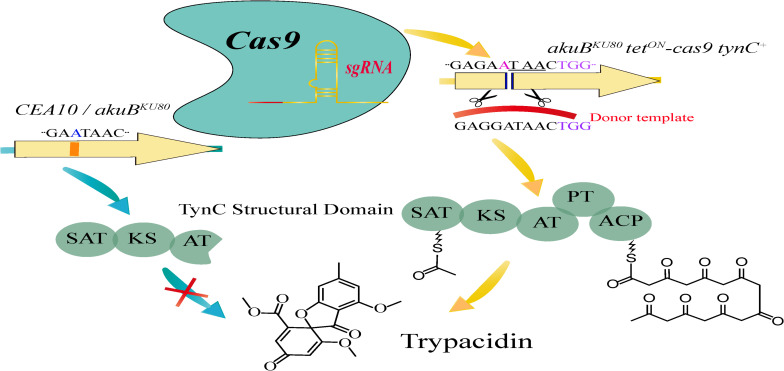
Overview of the CRISPR/Cas9-based tool for functional reconstitution of the *Aspergillus fumigatus* secondary metabolite (trypacidin) gene cluster.

In a previous study, Frisvad et al. (2009) analyzed and detected trypacidin in 30 out 40 *A. fumigatus* strains of different origins, including the clinical isolate strain Af293. The remaining 10 strains from which they did not isolate trypacidin, included a second clinical isolate and lab strain CEA10 (Frisvad et al., 2009). Based on these results, Weber et al. (2017) explored the mechanisms underlying the difference in trypacidin production and reconstituted the biosynthetic pathway of this compound by advanced genome editing in a non-producing strain. Initially, the area of surrounding a single nucleotide insertion in the polyketide synthase (PKS) coding gene *tynC* that potentially led to a frameshift and appearance of a premature stop codon in the CEA10 strain genome was sequenced from the *tynC* alleles of Af293 as well as CEA10 and its descendant strain CEA17 (Δ*akuBKU^80^*) (Throckmorton et al., 2016). The sequencing results identified a single adenosine base insertion at position 3881 of *tynC* in strain CEA10 and Δ*akuBKU^80^*. This insertion resulted in a premature stop codon, thus eliminating the predicted acyl carrier protein (ACP) and the product template (PT) domains that are essential for the catalytic function of the PKS. The authors then performed single nucleotide editing by the traditional gene substitution method and detected trypacidin in the complemented strain. However, this is a cumbersome multistep method. Therefore, an alternative strategy using a CRISPR/Cas9-based tool was adopted to reconstitute trypacidin production (Figure 3). Weber et al. (2017) initially constructed a recombinant Cas9 expression cassette and integrated it into strain CEA17 Δ*akuB pyrG* ^+^ (*akuB*^KU80^ *tet*^ON^-*cas9*). They then developed a plasmid containing a split-marker (Kuck and Hoff, 2010) and gRNA (*pJW split-ptrA tynC*), which was finally transformed into the same strain. By adding doxycycline to the fungal preculture before transformation, the expression of the Cas9 gene was induced to cause gene editing at the *tynC* locus. Subsequently, the plasmid *pJW split-ptrA tynC* coupled with donor DNA fragment were co-transformed into *akuB*^KU80^ *tet*^ON^-*cas9* via protoplast-mediated transformation (Weidner et al., 1998). The genomic DNA at the target site of selected positive transformants was sequenced. LC-MS (Mattern et al., 2015) was then applied to analyze stationary phase cultures, and a RNeasy Plant Mini kit was used to extract total RNA extraction. As expected, the single adenosine insertion was eliminated from the *tynC* locus of the *akuB*^KU80^ *tet*^ON^-*cas9 tynC ^+^* strain, and the presence of trypacidin as well as the *tynC* mRNA was detected. Thus, trypacidin was reconstituted through CRISPR/Cas9-mediated deletion of an adenosine insertion in the genome. Compared with the conventional strategy, the CRISPR/Cas9 gene editing system was demonstrated to be highly effective and a powerful tool in researching natural products from biosynthetic genes in *A. fumigatus*. This approach can also be helpful for the integration of fusion-tags and paves the way to exploit novel natural products derived from other filamentous fungi.

### CRISPR/Cas9-Based Genome Editing in *Glarea lozoyensis* Produces Pneumocandin B_0_

Pneumocandins are lipohexapeptides within the echinocandin family, and they potently impede fungal cell wall formation via non-competitive inhibition of β-(1, 3)-glucan synthases (Emri et al., 2013; Robbins et al., 2017). One of these compounds, pneumocandin B_0_, can be isolated from the industrial filamentous fungus *Glarea lozoyensis* and is used in the synthesis of the potent antifungal drug caspofungin (Emri et al., 2013; Balkovec et al., 2014). Because it has a strong inhibitory effect on invasive aspergillosis, caspofungin has been approved by the U.S. FDA for treating patients who are refractory or intolerant to standard therapy as well as for the main treatment of certain types of *Candida* infections (Leonard et al., 2007). Several researchers have explored the possibilities of improving pneumocandin B_0_ production because of its value in the pharmaceutical industry. However, owing to limitations and a lack of sophistication of traditional genetic tools, generation of a genetically engineered industrial *G. lozoyensis* strain has been impeded.

**FIGURE 4:**
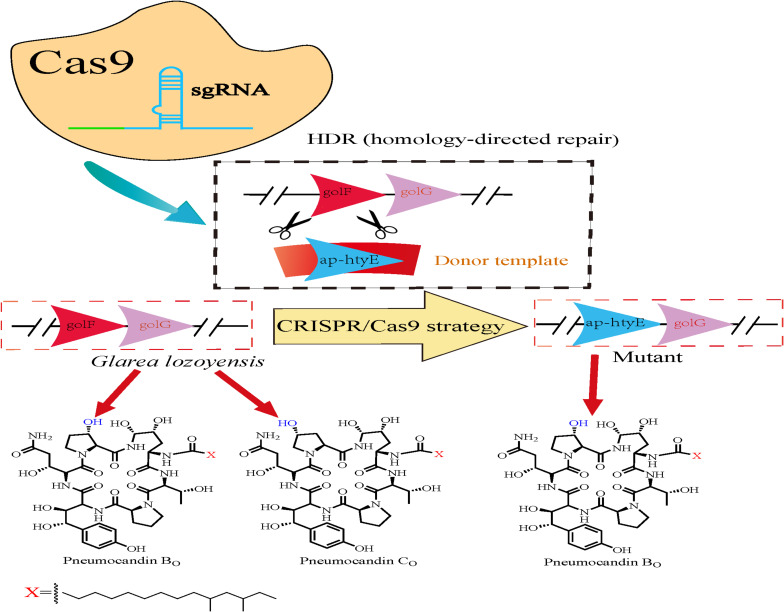
Schematic representation of gene replacement by the CRISPR/Cas9 strategy. The mutant retained the capability to produce pneumocandin B_0_, while the production of pneumocandin C_0_ was abolished.

Recently, Wei et al. (2020) utilized an efficient CRISPR/Cas9-based gene editing tool in *G. lozoyensis* SIPI1208 to significantly enhance the accumulation of pneumocandin B_0_ (Figure 4). Specifically, this strategy was used to replace *GloF* with *Ap-HtyE* (proline hydroxylase, which is responsible for pneumocandin and echinocandin B biosynthesis, separately) in *G. lozoyensis* using CRISPR/Cas9 system-mediated homology-directed repair (HDR), thus changing the ratio of pneumocandin B_0_ and pneumocandin C_0_ products (which constitute a pair of isomers). They initially designed a protospacer sequence targeted to *gloF* and constructed the pAgG-sgRNA-*gloF* plasmid to perform *gloF* gene editing. Thereafter, donor DNA was ligated into the linearized plasmid to construct the final replacement plasmid pAgG-sgRNA-*gloF-ap-htyE* in order to create a knock-in mutant, *ap-htyE*. After *Agrobacterium tumefaciens*-mediated transformation, a series of experimental verifications confirmed that the genomic DNA of *ap-htyE* was knocked in correctly (Zhang et al., 2003). Based on combined reversed-phase-HPLC and normal phase-HPLC analyses of the fermentation extracts (Osawa et al., 1999), it was found that PC_0_ was not present in the final fermented product of the knock-in strain, as compared to 33.5% PC_0_ in the original strain *G. lozoyensis*. These results illustrated that the ability to produce PC_0_ was abolished in the gene-edited strain generated using the CRISPR/Cas9 system, thus enabling increased industrial production of PB_0_ (Wei et al., 2020). In summary, the CRISPR/Cas9-based gene editing method can efficiently manipulate genes in *G. lozoyensis* and thus enabled for the development of production of other secondary metabolites with similar characteristics.

### CRISPR-Mediated Activation of *micA* Synthetase Gene Increases Microperfuranone Production

The CRISPR-mediated strategy mentioned earlier is not only capable of genetic modification but can also modulate transcription by fusing dCas9 to transcriptional repression and activation domains (i.e., through CRISPRa and CRISPRi). In contrast to the type-II CRISPR/Cas system, CRISPRa and CRISPRi do not require DSBs or donor DNA. The core part of achieving transcriptional activation is the link between the activation domain and dCas9, which then forms a complex with a sgRNA that includes an editable 20-nucleotide sequence complementary to the target site in the gene regulatory region (Chavez et al., 2015; Tak et al., 2017). Furthermore, by concurrently expressing multiple gRNAs, several genes can be simultaneously activated with multiplexed CRISPRa (McCarty et al., 2020). A previous study demonstrated that CRISPRa is a simple and universally applicable technology, by successfully employing it to increase the transcriptional efficiency of *Myxococcus xanthus* secondary metabolites (Peng et al., 2018). Nevertheless, the application of CRISPRa to the transcriptional regulation of biosynthetic pathways of secondary metabolites in filamentous fungi could be exploited further.

**FIGURE 5:**
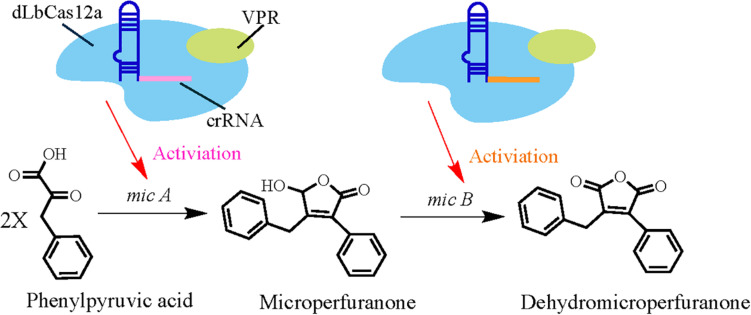
Schematic representation of CRISPRa-mediated increases in the production of microperfuranone and discovery of the mic cluster product, dehydromicroperfuranone.

In a recent report, researchers developed a CRISPRa system for *A. nidulans* for the first time in a filamentous fungus (Roux et al., 2020). They used dCas9 protein fusion to VP64-p65-Rta (VPR, a fusion of three activation domains) and tested whether it leads to strong activation (Chavez et al., 2015). Initially, CRISPR/dLbCas12a-VPR and CRISPR/dSpCas9-VPR-based systems were constructed and tested to exploit their utility. Thereafter, by delivering the four-crRNA array on an AMA1-pyroA vector and the sgRNA in a single AMA1-*pyrG* vector in two separate systems, they evaluated the multiplexing capability of the dCas12a- and dCas9- driven systems. Ultimately, the CRISPR/dLbCas12a-based CRISPRa system was chosen to probe the activation of biosynthetic genes in *A. nidulans* for its potential in BCG activation and accelerating the discovery of secondary metabolites (Fonfara et al., 2016). Using this CRISPRa system, Sanson et al. (2018) targeted the *micA* gene (which is related to microperfuranone biosynthesis) with multiple crRNAs in order to improve the likelihood of achieving strong activation (Figure 5). Following previously designed guidelines, region 119–303 bp and 139–324 bp upstream of the *micA* transcription start site (Jensen, 2018) were targeted with four-crRNAs to explore the utility of CRISPRa. Then, linearized pCRI001-3 vectors were used to promote homologous recombination followed by polyethylene glycol (PEG)-calcium-based protoplast transformation (Lim et al., 2012). Liquid chromatography coupled with diode array detector and mass spectrometer (LC-DAD-MS) analysis of the media extracts from all CRISPRa transformants showed that the accumulation of microperfuranone was improved when compared with control strains. In addition, the dCas12a-driven system was also utilized to explore simultaneous activation of multiple genes in the *mic* cluster by adopting the above-mentioned method, but with the addition of two crRNA arrays delivered into the strains. Analysis of *A. nidulans* culture extracts by LC-DAD-MS indicated that microperfuranone levels decreased in the multiplexed-CRISPRa strains and also revealed a new compound. Subsequent LC-MS/MS and NMR analysis identified this new compound as dehydromicroperfuranone, which is the metabolic product of the *mic* cluster (Figure 5). Thus, these results demonstrate that CRISPRa is efficient in multi-gene activation of BGCs in *A. nidulans* and can therefore be used to achieve higher throughput natural production of known and novel bioactive secondary metabolites in filamentous fungi.

## Conclusion and Perspectives

Filamentous fungi, like other eukaryotes, are extensively used in industrial and pharmaceutical production owing to their ability to secrete a plethora of hydrolytic enzymes, polyunsaturated fatty acids, and, more importantly, bioactive small molecules, including antibiotic agents. Furthermore, filamentous fungi are the most compatible heterologous hosts for expression of compounds such as *Penicillium citrinum* nuclease P1 in *A. niger* and fungal BGCs in *Fusarium graminearum*, owing to their lack of a requirement of intron-removal or codon optimization (He et al., 2018; Chen et al., 2019; Nielsen et al., 2019). With advances in genome sequencing and phylogenetic sleuthing, large repertoires of cryptic or silent secondary metabolite biosynthetic gene clusters are being uncovered. However, the difficulties in genetic manipulation have traditionally impeded secondary metabolite molecular studies on filamentous fungi. Therefore, a powerful and comparatively simple genetic technique for overcoming the obstacle of activating these silent secondary metabolite biosynthetic gene clusters is urgently needed. CRISPR/Cas9 is based on a conservative immune defense mechanism found in bacteria and archaea and has been developed as a convenient and flexible technique for genome editing. The CRISPR/Cas9 genome editing technology has shown great promise in revolutionizing the field of fungal research. The CRISPR/Cas9 genome editing system was first introduced into *Saccharomyces cerevisiae* (DiCarlo et al., 2013). Subsequently, Liu et al. (2015) applied the CRISPR/Cas9 genome editing system to *Trichoderma reesei*; Matsu-Ura et al. (2015) and Nodvig et al. (2015) applied this system to the model fungal *Neurospora crassa* and *A*. *nidulans*, respectively. Since then, the CRISPR/Cas9 genome editing system has found wide applications in genetic alteration of many filamentous fungi. These studies strongly confirm that the CRISPR/Cas9 gene-editing system will undoubtedly bring great possibilities for the discovery of new secondary metabolites, on account of its straightforward design, high efficiency, and versatility.

Compared with genome editing approaches that utilize protein-guided programmable nucleases such as ZFN and TALEN-based genome editing tools, nucleic acid-guided nucleases such as that primarily utilized in the CRISPR/Cas9 system have several advantages for genetic engineering to produce secondary metabolites. One advantage is that the CRISPR/Cas9 system can potentially edit almost all genes containing a PAM in their target sequences owing to its simplicity and modularity. Only two components are required in this system: a Cas9 endonuclease and sgRNA (Song et al., 2019). Indeed, there remain challenges and limitations to utilize this approach, such as off-target effects and the need to perform precise editing. As research advances, a well-designed gRNA sequence and specific Cas9 variants can be developed to avoid off-target effects, as can control the amount of intracellular Cas9 or enhance Cas9 for higher specificity through protein engineering (Cho et al., 2014; Tong et al., 2019). For example, Pohl et al. (2016) adopted a strategy for assembling the Cas9-gRNA complex *in vitro* and co-transformed it with a donor DNA into *Penicillium chrysogenum*, which proved to be effective in reducing off-target effects. With regard to precise editing, the biggest hurdle is the native NHEJ that intensely affects the efficiency of HDR despite the presence of a homologous template (Tong et al., 2019). By employing the inhibitor Scr7, Maruyama et al. (2015) successfully targeted the DNA ligase IV responsible for the NHEJ pathway, causing suppression of the native NHEJ activity, thus improving the efficiency of precise genome editing. Additionally, several advanced strategies like CRISPRi and FokI-dCas9 have been applied to optimize the Cas9 system to perform more precise gene-editing (Guilinger et al., 2014; Kao and Ng, 2017).

The potential of fungal biosynthetic capabilities in generating secondary metabolites seems virtually unlimited. Researchers have continually explored and developed secondary metabolites from filamentous fungi, with CRISPR/Cas9 technologies now accelerating progress. The powerful advantages of the CRISPR/Cas9 system in studies on filamentous fungal secondary metabolites reduce the application of selective markers. Most secondary metabolites are regulated by gene clusters in filamentous fungi. When three or more genes need to be manipulated at the same time, multiple selection markers were required and thus developed for filamentous fungi. The CRISPR/Cas9 system can edit multiple genes at the same time, and it is possible to obtain mutants with multiple site mutations in a single transformation, which greatly improves the efficiency of genome editing in studies on secondary metabolites of filamentous fungi. However, relatively few reports about the “actual applications” of the CRISPR/Cas9 system in the production of secondary metabolites can be obtained. Thus, the application is still in its initial stages, as most of the research focus is on aspects such as assessing the feasibility of CRISPR/Cas9 systems in fungi (Tong et al., 2019). Apart from CRISPR/Cas9 genome editing strategies, alternative methods, such as CRISPRi and CRISPRa, which do not depend on DSBs, were rarely reported in filamentous fungi, especially with respect to secondary metabolite production. So far, only one application of the CRISPRa technique being used to increase the secondary metabolite contents of a filamentous fungus has been reported (Roux et al., 2020). CRISPRi has not been used in filamentous fungi, but has been successfully applied in other fungi (Roman et al., 2019). In addition, prime editing, an emerging and precise genome editing method that expands the scope of CRISPR genome editing, with few byproducts and without requiring DSBs or donor DNA templates, has attracted great attention (Anzalone et al., 2019). Consequently, this method has potential value in research on secondary metabolites from filamentous fungi. Related technologies and applications of the CRISPR/Cas9 possess great potential and will play a greater role in the discovery of new secondary metabolites and engineering of the strains that produce them in the near future.

In summary, CRISPR/Cas9-based genome editing technology still requires development and improvement in genetic modification of secondary metabolites in filamentous fungi, and the general scope of applications can be expanded further. It is premature to declare that the CRISPR/Cas9 technique is accelerating the metabolic engineering of filamentous fungi for secondary metabolites. However, with the development of full genomes available and metabolomics, knowledge of the secondary metabolite biosynthetic gene clusters of filamentous fungi together with exploitation of CRISPR/Cas9 approaches will help overcome current limitations in increasing production of secondary metabolites. Such advances will also promote the discovery of new bioactive compounds.

## Author Contributions

GL: conceptualization and writing—original draft preparation. CJ: writing—review and editing and conceptualization. XC and YT: project administration. YD: supervision. BZ: supervision and funding acquisition. BH: writing—review, editing, and funding acquisition. All authors contributed to the article and approved the submitted version.

## Conflict of Interest

The authors declare that the research was conducted in the absence of any commercial or financial relationships that could be construed as a potential conflict of interest.
